# The Impact of Obesity and Insulin Resistance on Iron and Red Blood Cell Parameters: A Single Center, Cross-Sectional Study

**DOI:** 10.4274/Tjh.2012.0187

**Published:** 2014-03-05

**Authors:** Esma Altunoğlu, Cüneyt Müderrisoğlu, Füsun Erdenen, Ender Ülgen, M. Cem Ar

**Affiliations:** 1 İstanbul Training and Research Hospital, Department of Internal Medicine, İstanbul, Turkey; 2 Istanbul University, Cerrahpaşa Medical Faculty, Department of Internal Medicine, Division of Haematology, Istanbul, Turkey

**Keywords:** obesity, insulin resistance, Anemia, inflammation

## Abstract

**Objective:** Obesity and iron deficiency (ID) are the 2 most common nutritional disorders worldwide causing significant public health implications. Obesity is characterized by the presence of low-grade inflammation, which may lead to a number of diseases including insulin resistance (IR) and type 2 diabetes. Increased levels of acute-phase proteins such as C-reactive protein (CRP) have been reported in obesity-related inflammation. The aim of this study was to investigate the impact of obesity/IR on iron and red blood cell related parameters.

**Materials and Methods:** A total of 206 patients and 45 control subjects of normal weight were included in this cross-sectional study. Venous blood samples were taken from each patient to measure hemoglobin (Hb), serum iron (Fe), iron-binding capacity (IBC), ferritin, CRP, fasting blood glucose, and fasting insulin. Body mass index (BMI) and waist-to-hip ratio (WHR) were calculated for each patient. IR was determined using the HOMA-IR formula.

**Results:** Subjects were divided into 3 groups according to BMI. There were 152 severely obese (BMI: 42.6±10.1), 54 mildly obese (BMI: 32.4±2.1), and 45 normal-weight (BMI: 24.3±1.3) patients. Hb levels in severely obese patients and normal controls were 12.8±1.3 g/dL and 13.6±1.8 g/dL, respectively. We found decreasing Fe levels with increasing weight (14.9±6.9 µmol/L, 13.6±6.3 µmol/L, and 10.9±4.6 µmol/L for normal controls and mildly and severely obese patients, respectively). Hb levels were slightly lower in patients with higher HOMA-IR values (13.1±1.5 g/dL vs. 13.2±1.2 g/dL; p=0.36). Serum iron levels were significantly higher in the group with low HOMA-IR values (13.6±5.9 µmol/L vs. 11.6±4.9 µmol/L; p=0.008). IBC was found to be similar in both groups (60.2±11.4 µmol/L vs. 61.9±10.7 µmol/L; p=0.23). Ferritin was slightly higher in patients with higher HOMA-IR values (156.1±209.5 pmol/L vs. 145.3±131.5 pmol/L; p=0.62).

**Conclusion:** Elevated BMI and IR are associated with lower Fe and hemoglobin levels. These findings may be explained by the chronic inflammation of obesity and may contribute to obesity-related co-morbidities. People with IR may present with ID without anemia.

## Abstract (tr)

**Amaç:** Dünyada en sık görülen beslenme bozukluklarından olan obezite ve demir eksikliği önemli halk sağlığı sorunlarına yol açmaktadır. Kronik, düşük dereceli inflamasyon ile karakterize olan obezite insülin direnci ve tip 2 diyabetes mellitus gibi ciddi hastalıklara zemin hazırlar. Obezite ile ilişkili inflamasyonun C reaktif protein (CRP) ve bazı sitokinler gibi akut faz yanıtı oluşturan proteinlerin düzeyinde artışa neden olduğu gösterilmiştir. Bu çalışmada obezite ve insülin direncinin demir ve eritrosit ile ilişkili parametreler üzerindeki etkisinin araştırılması amaçlanmıştır.

**Gereç ve Yöntemler:** Bu tek merkezli, kesitsel çalışmaya obezite polikliniğine başvuran 206 hasta ve 45 normal kilolu sağlıklı kontrol dahil edilmiştir. Hastalardan alınan venöz kan örneklerinde hemoglobin (Hb), demir, demir bağlama kapasitesi (DBK), ferritin, CRP, açlık şekeri ve insülin düzeyleri çalışılmıştır. Ayrıca çalışmaya alınan her hastanın vücut kütle indeksi (VKİ), bel/kalça oranı (BKO) hesaplanmış, insülin direnci HOMA-IR formülü ile bulunmuştur.

**Bulgular:** Hastalar VKİ’lerine göre 3 gruba ayrıldı. Buna göre ileri derecede obez olan 152 hasta (VKİ: 42,6±10,1), hafif obez 54 hasta (VKİ: 32,4±2,1) ve normal kilolu 45 kontrol (VKİ: 24,3±1,3) vardı. İleri derecede obezlerle karşılaştırıldığında normal kilolu hastalarda Hb düzeyleri daha yüksek (sırasıyla 12,8±1,3 g/dL ve 13,6±1,8 g/dL) saptandı. Serum demir düzeylerinin artan kilo ile birlikte azaldığı görüldü (normal, hafif ve ileri derecede kilolu hastalarda sırasıyla 14,9±6,9 µmol/L, 13,6±6,3 µmol/L ve 10,9±4,6 µmol/L) HOMA-IR değeri yüksek hastalarda düşük olanlara nazaran Hb düzeyleri hafif azalmış bulundu (13,1±1,5 g/dL’ye karşılık 13,2±1,2 g/dL; p=0,36). Serum demir düzeyleri düşük HOMA-IR’lı hastalarda anlamlı olarak daha yüksekti (13,6±5,9 µmol/L’ye karşılık 11,6±4,9 µmol/L; p=0,008). DBK her iki grupta da benzerdi (60,2±11,4 µmol/L’ye karşılık 61,9±10,7 µmol/L; p=0,23). Ferritin düzeyleri yüksek HOMA-IR’lı grupta anlamlı olmasa da daha yüksek bulundu (156,1±209,5 pmol/L’ye karşılık 145,3±131,5 pmol/L; p=0,62).

**Sonuç:** Artmış VKİ ve insülin direnci, düşük hemoglobin ve serum demir düzeyleri ile ilişkili bulunmuştur. Bu bulgular kısmen obezitede görülen düşük dereceli inflamasyon ile açıklanabilir ve obezite ile ilişkili ek hastalıkların oluşmasına katkı sağlıyor olabilir. İnsülin direnci bulunan hastalarda anemi gözlenmeksizin subklinik demir eksikliği görülebilir.

## INTRODUCTION

Obesity and iron deficiency (ID) are 2 of the most common nutritional disorders worldwide [1]. ID, in developed countries, is the most common nutritional deficiency and has been linked to obesity in adults and children [2]. The association between iron status and obesity is one that should be explored further, as obesity and ID are diseases that continue to evolve globally, and both have significant public health implications [3]. The global incidence of obesity has increased dramatically over the past 50 years. Currently more than 1 billion people are thought to have a body mass index (BMI) of more than 30 kg/m2, and the number is expected to increase dramatically over the next 30 years [4]. The prevalence of ID and iron deficiency anemia (IDA) is highest in the developing world; however, suboptimal iron status continues to exist in the developed countries. Epidemiological studies have shown that the prevalence of anemia increases with age [5]. Among micronutrients, iron plays a major role not only for hemoglobin synthesis alone, but also for oxidative metabolism and energy production. ID and IDA have been shown to underlie important public health issues; diminished iron reserves affect cognitive development and behavior, energy metabolism, immune function, bone health, and work capacity in humans [6].

The inverse correlation between plasma iron and adiposity in children and adolescents was recently reported by Pinhas-Hamiel et al. They showed that low iron levels were present in 38.8%, 12.1%, and 4.4% of obese, overweight, and normal-weight children, respectively [7]. Obesity is characterized by the presence of low-grade inflammation and the risk of developing a number of chronic diseases, such as insulin resistance (IR) and type 2 diabetes. Obesity-related inflammation increases plasma levels of many acute-phase proteins such as C-reactive protein (CRP), hepcidin, and several cytokines [8]. We hypothesized that IR and obesity may be associated with decreased hemoglobin and changes in the iron parameters, including serum iron levels, iron-binding capacity (IBC), and serum ferritin. Obesity-related inflammation is expected to result in reduced hemoglobin and serum iron levels in obese adults and subjects with IR. The purpose our study was to examine the relation among hemoglobin, serum iron, total IBC, CRP, and IR in obese patients and compare this to normal-weight adults. 

## MATERIALS AND METHODS

This cross-sectional study included 251 subjects (203 females and 48 males) comprising 206 patients, with no established chronic or hematologic diseases, and 45 normal-weight healthy controls without any prior personal history of obesity or diet. None of the patients had undergone gastric bypass surgery or an intervention for the surgical treatment of obesity. Menstrual cycles and reproductive history were obtained in premenopausal women. Women with menstrual irregularities were excluded. People on iron therapy or consuming dietary supplements or vitamins containing iron, and subjects having received non-steroidal anti-inflammatory drugs 48 h prior to blood sampling, were also exempted. None of the included subjects had a history of blood donation or transfusion. Neither patients nor the healthy controls were regular alcohol drinkers. All patients were weighed using a digital weighing scale with light clothing on, and without shoes. Their heights were measured standing on a fixed stadiometer. BMI was calculated by dividing body weight in kilograms by body height squared in meters and patients were divided according to the World Health Organization recommended cut-off points into 5 groups: normal weight (BMI of <25 kg/m2), overweight (BMI of 25 to <30 kg/m2), mildly obese (BMI of 30 to <35 kg/m^2^), moderately obese (BMI of 35 to <40 kg/m^2^), and severely obese (BMI of >40 kg/m2). Waist circumference (WC) and hip circumference (HC) were also measured using a flexible measuring tape and waist-to hip ratio (WHR) was then calculated. All subjects fasted for at least 12 h before blood sampling for biochemical analysis. Fasting blood glucose levels were measured spectrophotometrically using the Abbot Aeroset 2.0 (Abbot Diagnostic, USA). Serum iron was measured by the ferrozine method. Serum iron of less than 8.95 µmol/L indicated ID based on the reference ranges (normal range: 8.95-30.43 µmol/L) provided by the manufacturer. Ferritin was measured using the chemiluminescence method and values of less than 22.47 pmol/L (normal range: 22.47-633.65 pmol/L) were accepted as ID. The analysis of CRP was performed by immunoturbidity. Hemoglobin and hematocrit were measured with the Advia 2120 Siemens blood counter. According to World Health Organization guideliness the standard values of grading of anemia are <12 g of hemoglobin in female and <13 g of hemoglobin in male. Insulin was measured by the electro-chemiluminescence immunoassay method on a Roche-Hitachi E 170. IR 

was calculated using the homeostasis model assessment formula: HOMA-IR =(fasting insulin (mU/L) x glucose (mmol/dL)/22.5. The study was conducted in accordance with the Helsinki Declaration and rules of Good Clinical Practice. It was approved by the local ethics committee.

**Statistics**

Statistical analysis was performed using SPSS 17.00 for Windows. Baseline data were expressed as means±standard deviations. To analyze differences between groups we used the independent samples t-test. A value of p<0.05 was considered as significant. 

## RESULTS

The information about age, sex, and body measurements of the 251 subjects are given in Table 1. On the basis of BMI, 3 groups were identified: there were 152 severely obese (BMI: 42.6±10.1), 54 mildly obese (BMI: 32.4±2.1), and 45 normal-weight (BMI: 24.3±1.3) patients. As expected, severely and mildly obese patients had significantly higher values for BMI, WC, HC, and WHR when compared to normal controls. CRP levels were higher in the obese group, although without statistical significance. Hemoglobin and hematocrit levels were significantly higher in people with normal weight when compared to the severely obese (13.6±1.1 g/dL vs. 12.8±1.3 g/dL, p=0.015; 41.0±3.8% vs. 38.5±3.6%, p=0.007, respectively) (Table 2). This may partly be explained by the lower female-to-male ratio in the normal-weight healthy controls in comparison to the severely obese group (0.64 vs. 0.90). Hemoglobin and hematocrit levels were higher in both normal and obese males than normal and obese females. Hemoglobin values were 14.2±0.8g/dl vs 14.7±1.1g/dl p=0.15; 13.1±0.6g/dl vs12.7±1.2g/dl p=0.02 respectively. Hematocrit values were 44; 0±42.6% vs 44.0±43.1% p=0.98; 39.0±2.4% vs. 38.1±3.5% p=0.37, respectively (Table 3). Although there was no significant association between ferritin levels and the degree of obesity, ferritin values were higher in the non-obese group. Serum iron levels were measured to be significantly higher in people with normal weight than in the obese subjects (p=0.001). An opposite trend was observed for IBC. In comparison to obese subjects, those with normal weight had lower levels of IBC. The cut-off value for HOMA-IR was accepted as 2.5. There were 86 and 165 patients with HOMA-IR levels of ≤2.5 and >2.5, respectively. Hemoglobin levels in people with higher IR (as indicated by increased HOMA-IR) were slightly lower as compared to those with lower HOMA-IR values (13.1±1.5 g/dL vs. 13.2±1.2 g/dL; p=0.36). No difference in hematocrit levels was observed between patients with >2.5 and ≤2.5 HOMA-IR values (39.8±3.7% vs. 39.1±4.3%; p=0.20). Serum iron levels were significantly higher in the group with HOMA-IR values of ≤2.5 (11.6±4.9 µmol/L vs. 13.6±5.9 µmol/L; p=0.008). IBC was found similar in both groups (60.2±11.4 µmol/L vs. 61.9±10.7 µmol/L; p=0.23). Ferritin levels were slightly higher in the group with >2.5 HOMA-IR values (156.1±209.5 pmol/L vs. 145.3±131.5 pmol/L; p=0.62). CRP levels in the group with high HOMA-IR values were higher than those in the group with low HOMA-IR values, but the difference was not significant (11.1±10.1 nmol/L vs. 11.3±11.4 nmol/L; p=0.83). Demographic and hematological characteristics of the groups according to IR are shown in Table 3.

## DISCUSSION

Obesity and related complications as well as ID are 2 major issues that affect significant proportions of the global population [9]. This is of considerable concern for the well-being of the population given that overweight and obese people are at increased risk for co-morbidities, functional decline, impaired quality of life, increased use of health care resources, and increased mortality. Iron plays a vital role in hemoglobin production and erythrocyte maturation. Two of the most common causes of anemia, IDA and the anemia of chronic inflammation, result from abnormalities in iron homeostasis [10]. Iron homeostasis in the body is controlled by a very complex mechanism, the main components of which are erythropoietic activity, hypoxia, iron stores, and inflammation [6].

However, iron may also function in the maintenance of body weight and composition, as a number of studies have suggested an association between iron status and obesity. The first such study, published in 1962 by Wenzel et al., demonstrated significantly lower serum iron concentrations in obese adolescents in comparison to normal controls [11]. Subsequently, Selzer and Mayer reported similar findings in 1963 [12]. More recently, in a cross-sectional study, overweight Israeli children and adolescents had lower iron status compared with normal-weight individuals [7]. Data from NHANES III support these findings, as multivariate regression analysis determined that overweight American children were twice as likely to be iron deficient than normal-weight children and adolescents [13]. Similar associations have also been reported in adults [10]. 

Menzie et al. found significantly lower levels of serum iron and transferrin saturation in obese people when compared to non-obese adult volunteers. They reported that the obese and the non-obese subjects did not differ in total daily iron consumption but that fat mass was a significant negative predictor of serum iron level [14]. 

Our study resulted in similar findings. We found significantly lower serum iron levels in severely obese patients than in the mildly obese group, and the mildly obese group had levels that were lower than those of the normal controls. IBC levels were lower in normal-weight individuals compared to obese patients. The mechanism underlying the reduced iron status in obese individuals remains to be clarified. Iron depletion might result from the increased iron requirement of obese people because of their larger blood volume and/or their consumption of energy-dense, nutrient-poor foods [3,7,14]. Another cause of hypoferremia may be the chronic inflammation seen in obesity [15]. Hepcidin levels are usually anticipated to increase due to the low-grade inflammation together with other acute phase markers, including ferritin. However, ferritin levels were unexpectedly lower in our obese patient group when compared to normal controls. This may partly be explained by the disproportionately higher male-to-female ratio in the control group. On the other hand, low ferritin levels in our obese patient group might also reflect the low total iron body stores. Although IDA was one of the exclusion criteria of this study, we might have missed cases of occult ID due to low-iron diets, poor absorption, etc. as the study was of cross-sectional design and no bone marrow biopsies were performed to confirm iron body stores. Yanoff et al. found similar results and stated that the hypoferremia of obesity appears to be explained both by true ID and by inflammatory-mediated functional ID [15].

As previously stated, obesity is characterized by the presence of low-grade inflammation and the risk of developing a number of chronic diseases such as IR, impaired glucose tolerance, and type 2 diabetes [16]. As expected, we observed a higher rate of IR in people with higher BMIs. We found a negative correlation between IR and serum iron levels. In the group with high HOMA-IR, serum iron and hemoglobin levels were low and serum IBC and ferritin values were high. 

Insulin causes a rapid stimulation of iron uptake by faT-cells and hepatocytes. Reciprocally, iron interferes with insulin action in the liver [17,18]. In addition, iron is a potent pro-oxidant that increases cellular oxidative stress, causing inhibition of insulin internalization and action, which results in hyperinsulinemia, IR, and abnormal β-cell function through iron toxicity [16,20,21,22,23,24,25]. Furthermore, iron overload may lead to IR disorders such as the metabolic syndrome and type 2 diabetes [26,27]. This involvement appears to be bidirectional: on one hand, iron accumulation favors IR and thus contributes to pancreatic beta cell dysfunction and diabetes; on the other hand, IR seems to facilitate iron accumulation within the body [27]. 

Several studies have shown that IR is closely related to the total body iron stores [26]. Oxidative stress and inflammation are involved in the interplay between iron overload and IR [27]. Ferritin levels have been shown to correlate positively with blood glucose and fasting serum insulin and negatively with insulin sensitivity [28]. In our study, serum ferritin levels were higher in patients with IR. Ferritin is an index of body iron stores and is also an inflammatory marker. Increased serum ferritin concentrations and excessive iron can contribute to hyperinsulinemia and reduced insulin function [20,24]. High levels of serum ferritin have been suggested to be a component of IR. High ferritin levels in insulin-resistant patients are thought to be mainly the result of a chronic inflammatory state. Lee et al. recently reported a significant relationship between IR and ferritin levels [29]. 

The estrogen binding protein levels may be reduced with increasing adiposity with concomitant increase in insulin. Therefore levels of free estrogen may rise up which may cause suppression of erythropoesis in female [30]. In our study we found that both normal and obese female subjects had lower hemoglobin levels than male. This is in accordance with the knowledge that women have lower hemoglobin values than male subjects as determined by reference interwals of WHO. CRP values were higher in this group. Adipose tissue, especially visceral adipose tissue, releases pro-inflammatory cytokines such as interleukin-6, tumor necrosis factor-α, and plasminogen activator inhibitor-I, which lead to increased plasma levels of acute-phase proteins such as CRP [10,30,31]. Inflammation has been reported to be mild, but it can promote anemia [32]. The low-grade inflammation induced by the aforementioned cytokines may contribute to the development of obesity-associated anemia, which is characterized by hypoferremia and high to normal serum ferritin levels. We used CRP as an inflammatory marker in this study. CRP levels were increased in correlation with BMI, although the association was not statistically significant. However, earlier studies showed a strong association between high levels of BMI and elevated CRP as a surrogate marker of low-grade inflammation [9,15]. Proinflammatory cytokine release might lead to the increased production and secretion of hepcidin from liver and adipose tissue [33,34]. Hepcidin inhibits intestinal iron absorption and sequesters iron within the macrophages, thereby restricting iron availability for erythrocyte production by inducing hypoferremia [24]. Recent studies have shown that dietary iron absorption is impaired in obese individuals despite adequate dietary intake and bioavailability [31,35]. 

Limitations of our study include the lack of additional tests required for evaluation of iron status, such as transferrin receptor concentration, reticulocyte count, hepcidin levels, and bone morrow iron stores. Bone morrow aspirates for stainable iron are sometimes required to confirm ID in obese subjects with high IBC. As our aim was to investigate the hematological parameters in patients with IR, we did not evaluate hepcidin and the aforementioned parameters. Another limitation is that this study was cross-sectional in design and therefore no conclusions regarding causal relationships could be drawn. The duration of obesity could not be calculated in our study population. 

Although obese patients had lower values for iron, they were not anemic. Given the cross-sectional design of this study, subjects were not followed to determine whether they would develop IDA over time.

## CONCLUSION

Increasing BMI has been associated with low serum iron and hemoglobin as well as elevated serum ferritin levels. These findings may be explained by the low-grade chronic inflammation of obesity and have been implicated in many obesity-related problems, such as IR. Subjects with IR may have subclinical abnormalities in iron status without anemia. Thus, routine screening for serum ferritin and serum iron in people with IR and obese patients should be considered to assess the body iron store and the risk for developing anemia. IR should be included in the differential diagnosis of hyperferritinemia. We recommend close monitoring of the iron status for people with IR and/or obesity who are on strict diet programs since hypoferremia appears to be the result of both true ID and the inflammatory-mediated functional ID. The patients with identified ID should receive iron supplements. 

## CONFLICT OF INTEREST STATEMENT

The authors of this paper have no conflicts of interest, including specific financial interests, relationships, and/ or affiliations relevant to the subject matter or materials included. 

## Figures and Tables

**Table 1:**
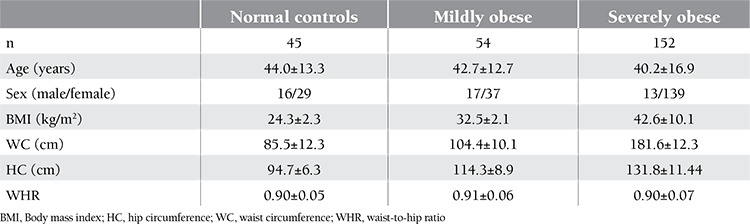
Age, sex, and body measurements of the subjects

**Table 2:**
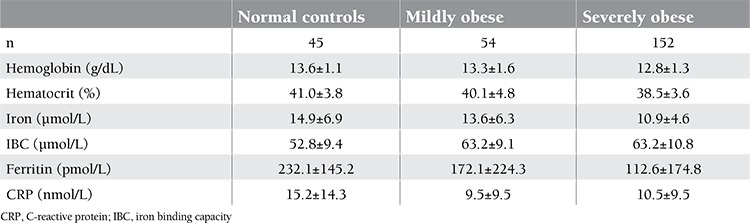
Laboratory characteristics of the patients according to BMI

**Table 3:**
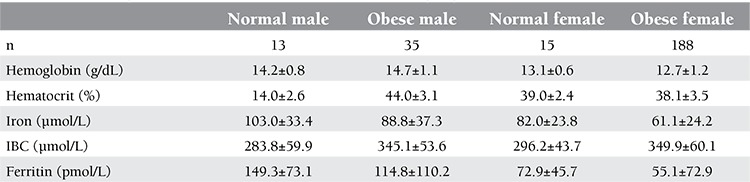
Haematologic laboratory characteristics of patients according to gender
